# Pterostilbene complexed with cyclodextrin exerts antimicrobial and anti-inflammatory effects

**DOI:** 10.1038/s41598-020-66031-8

**Published:** 2020-06-03

**Authors:** Yi Rong Ivan Lim, Philip M. Preshaw, Lum Peng Lim, Marianne Meng Ann Ong, Hai-Shu Lin, Kai Soo Tan

**Affiliations:** 1 Faculty of Dentistry, National University of Singapore, Singapore, Singapore; 2 National Dental Centre, Singapore, Singapore; 3 Department of Pharmacy, Faculty of Science, National University of Singapore, Singapore, Singapore

**Keywords:** Microbiology, Diseases

## Abstract

Resveratrol (RES) is a natural polyphenol with potential as an adjunctive therapeutic modality for periodontitis. However, its inferior pharmacokinetics and toxicity concerns about its commonly used solvent dimethyl sulfoxide (DMSO) hinder translation to clinical applicability. Our study aimed to investigate the comparative antimicrobial properties of RES and its analogues (pterostilbene [PTS], oxyresveratrol [OXY] and piceatannol [PIC]), utilizing 2-hydroxypropyl-β-cyclodextrin (HPβCD) as a solubiliser, which has a well-documented safety profile and FDA approval. These properties were investigated against *Fusobacterium nucleatum*, a key periodontal pathogen. PTS demonstrated the most potent antibacterial effects in HPβCD, with MIC > 60-fold lower than that of RES, OXY and PIC. In addition, PTS inhibited *F. nucleatum* biofilm formation. PTS exerted antimicrobial effects by eliciting leakage of cellular contents, leading to loss of bacterial cell viability. PTS also conferred immunomodulatory effects on *F. nucleatum-*challenged macrophages via upregulation of antioxidant pathways and inhibition of NF-κB activation. Given the superior antimicrobial potency of PTS against *F. nucleatum* compared to RES and other analogues, and coupled with its immunomodulatory properties, PTS complexed with HPβCD holds promise as a candidate nutraceutical for the adjunctive treatment of periodontitis.

## Introduction

Resveratrol (RES) is a polyphenolic compound that is found in various foods such as grapes, nuts, blueberries and red wine. Research on this compound experienced a significant upsurge after publications in the 1990s suggested a cardiovascular protective effect of RES resulting from wine consumption^1,2^ as well as describing a cancer chemopreventive activity^3^. Today, there is substantial literature documenting the wide range of biologic properties exhibited by RES. These include anti-ageing, anti-diabetic, anti-obesity, antioxidant, anti-inflammatory and antimicrobial effects^4–6^.

Periodontitis is a chronic inflammatory disease that affects the supporting structures of the teeth, leading to tooth mobility and tooth loss, with negative impacts on people’s quality of life. It results from the interaction of the host immune-inflammatory response with dysbiotic plaque biofilms in the subgingival environment^7^. Non-surgical periodontal therapy (professionally-delivered root surface debridement coupled with education to improve patient oral hygiene) is the cornerstone of initial periodontal treatment with established efficacy in reducing inflammation and improving clinical outcomes^8^. However, limitations in achieving ideal microbiological endpoints^9^, as well as the presence of other key risk factors such as smoking and diabetes can result in suboptimal clinical outcomes following treatment. Researchers have accordingly investigated the use of adjunctive treatments including antibiotics and host-modulatory therapies^10,11^ in the management of periodontitis, but concerns regarding risks of antibiotic resistance^12^ preclude their widespread use.

In light of these considerations, RES may hold promise as a natural adjunctive therapeutic in the treatment of periodontitis. Experimental studies in rats with ligature-induced periodontitis showed that administration of RES activated antioxidant pathways, decreased expression of pro-inflammatory cytokines and modulated alveolar bone loss^13–15^. Studies have also investigated the antimicrobial effects of RES against pathogens associated with periodontitis. However, these have yielded conflicting findings, with inhibition noted against *Aggregatibacter actinomycetemcomitans*, *Porphyromonas gingivalis*^16^ and *Fusobacterium nucleatum*^17^, while no antimicrobial effect was noted in other models^18^.

Despite the potential benefits of RES, there are some challenges in translating its use to clinical applicability. RES is an organic compound and is typically dissolved in organic solvents such as dimethyl sulfoxide (DMSO). However, DMSO exerts cytotoxic effects and therefore must be used at low concentrations^19^, with a recent study raising concerns about the toxic effects observed even at concentrations below 10% v/v^19^. Consequently, the need for a low concentration of DMSO limits the ability to dissolve critical amounts of RES required for meaningful biological activity.

To address this issue, cyclodextrins are a promising alternative solubility-enhancing excipient. Cyclodextrins are cyclic oligosaccharides, with the three most common natural cyclodextrins (α-, β-, γ-cyclodextrins) possessing 6, 7 and 8 linked glucopyranose units, respectively. The “cup-like” structure of cyclodextrins confers the ability to form inclusion complexes with drugs, usually in a 1:1 ratio^20^. Furthermore, because of their hydrophilic exterior encasing a hydrophobic cavity, cyclodextrins can function as transport vehicles delivering hydrophobic drug compounds to a target site^21^. As such, cyclodextrins have significant pharmaceutical utility, being widely used in the food and pharmaceutical industries^22^.

The differing number of linked D-glucopyranose units results in varying cavity sizes for the different types of cyclodextrins. With a cavity diameter of 6–6.5 Å, β-cyclodextrin has the most suitable cavity size for complexing with most small compounds^23^. Unfortunately, it also has lower aqueous solubility compared to α- and γ-cyclodextrin^21^. This is overcome via substitution of the hydroxyl groups for hydrophilic moieties such as hydroxypropyl groups. These modified, or “functionalized”, cyclodextrins have less intramolecular hydrogen bond formation, undergo less crystallization and thus have improved aqueous solubility. 2-hydroxypropyl-β-cyclodextrin (HPβCD) is an example of a modified cyclodextrin which enjoys improved aqueous solubility of ≥60% w/w^21^. Furthermore, HPβCD has proven *in vivo* safety with a low toxicology profile, having been evaluated in many safety studies^20^. It is a safe excipient for drug administration and has been given the United States Food and Drug Administration (FDA) approval for use in several marketed pharmaceutical products.

One key consideration is that changing the solvent for a particular drug may lead to different drug-solvent interactions, which in turn may affect the biological activity of the drug. For example, in an *in vitro* study, different antifungal potencies were noted when caspofungin and micafungin were dissolved in different solvents^24^. The inclusion complexes which cyclodextrins form with drug compounds have been shown to enhance not just solubility but also the physical and chemical stabilities of the drug^25,26^. As a result, cyclodextrins may confer beneficial potentiating properties when complexed with various drugs^27,28^. However, to the best of our knowledge, there has been no study to date investigating the effect, if any, on the antimicrobial properties of RES when dissolved in HPβCD.

**Figure 1:**
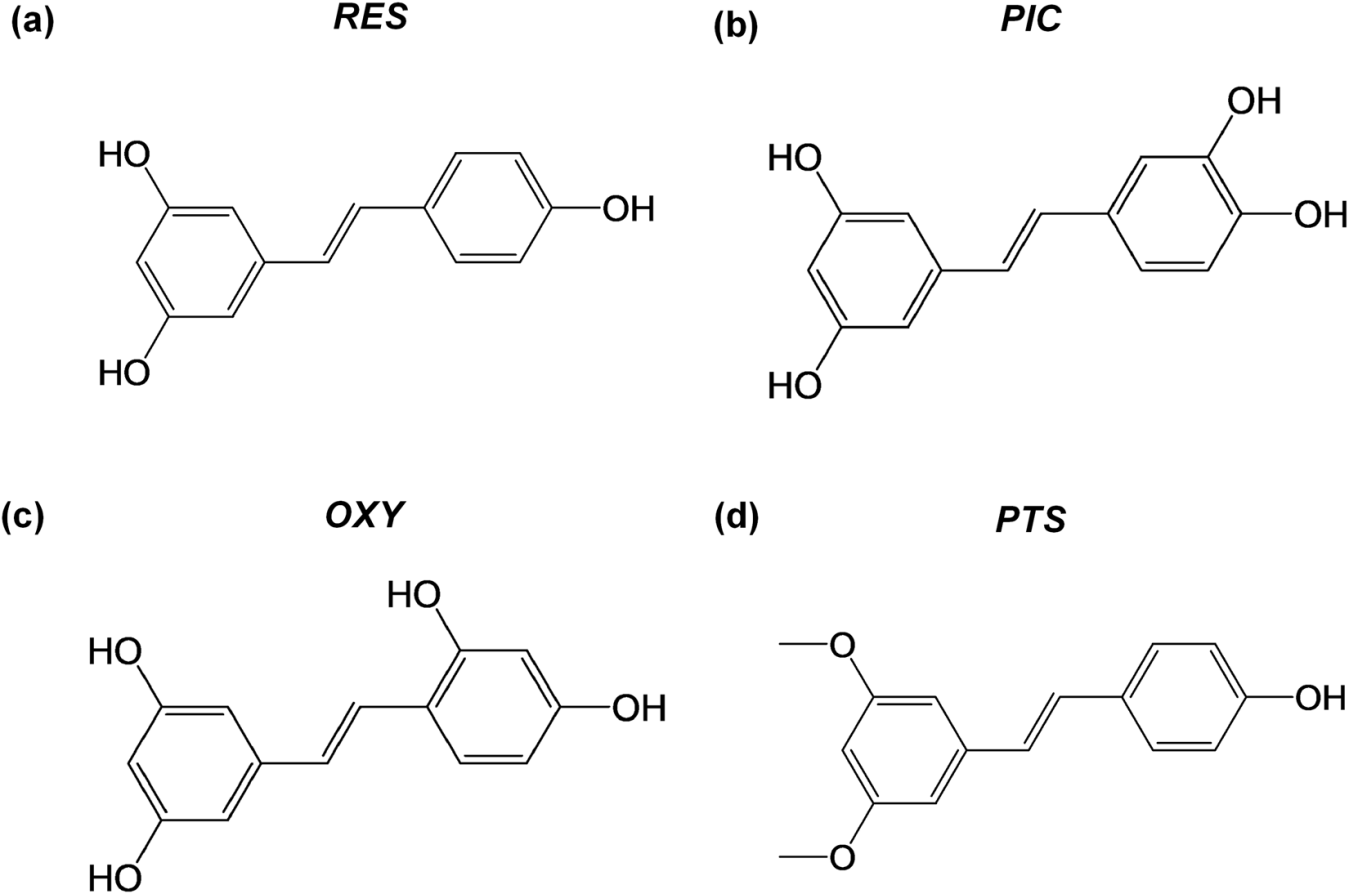
Chemical structures of (**a**) Resveratrol (RES), (**b**) Piceatannol (PIC), (**c**) Oxyresveratrol (OXY), and (**d**) Pterostilbene (PTS).

Other challenges in translating the utility of RES to clinical applications relate to its limited bioavailability and pharmacokinetic properties^4,29^. RES has a short half-life (about 8–14 minutes^30,31^), undergoes extensive phase II metabolism via glucuronide or sulphate conjugation of its hydroxyl groups^4,32^, and undergoes rapid elimination by the kidneys^33^. This has led to growing interest in other analogues of RES, which include piceatannol (PIC), oxyresveratrol (OXY) and pterostilbene (PTS) (Fig. 1).

Studies have demonstrated their superior pharmacokinetic properties compared to RES, such as more rapid absorption and slower elimination after intravenous administration^34^, and less susceptibility to conjugation metabolism^35,36^. When given as an oral solution in an animal model, PTS displayed superior metabolic stability, as evidenced by 5–6 times higher plasma exposure and mean transit time (MTT) compared to RES^37^. However, the antimicrobial properties of these analogues compared to RES remains an area yet to be investigated.

*F. nucleatum* is a key bridging organism in plaque biofilm and provides adhesins for other periodontal pathogens such as *P. gingivalis* to adhere^38^. *F. nucleatum* is also a strong instigator of inflammation. Given these important roles of *F. nucleatum* in building a dysbiotic oral microbial community and the ensuing inflammatory response, the aim of our study was to investigate the comparative antimicrobial properties of RES and its analogues against *F. nucleatum*, utilizing HPβCD as the vehicle.

## Results

### PTS in HPβCD exhibited enhanced antimicrobial properties

In this study, the minimum inhibitory concentration (MIC) of the test compound was defined as the minimum concentration of the test compound required to inhibit the growth of *F. nucleatum*. Minimum bactericidal concentration (MBC) was defined as the minimum concentration required to kill *F. nucleatum*. Table 1 shows the MIC and MBC values for RES, OXY, PIC and PTS when dissolved in HPβCD and DMSO.

**Table 1:** MIC and MBC of RES, OXY, PIC and PTS against *F. nucleatum* when dissolved in HPβCD or DMSO.

Compound	Solvent	MIC (mg/ml)	MBC (mg/ml)
RES	HPβCD	> 1.25	> 1.25
	DMSO	0.15	> 0.15
OXY	HPβCD	> 1.25	> 1.25
	DMSO	0.15	> 0.15
PIC	HPβCD	> 1.25	> 1.25
	DMSO	> 0.15	> 0.15
PTS	HPβCD	0.02	0.04
	DMSO	> 0.15	> 0.15

**Figure 2:**
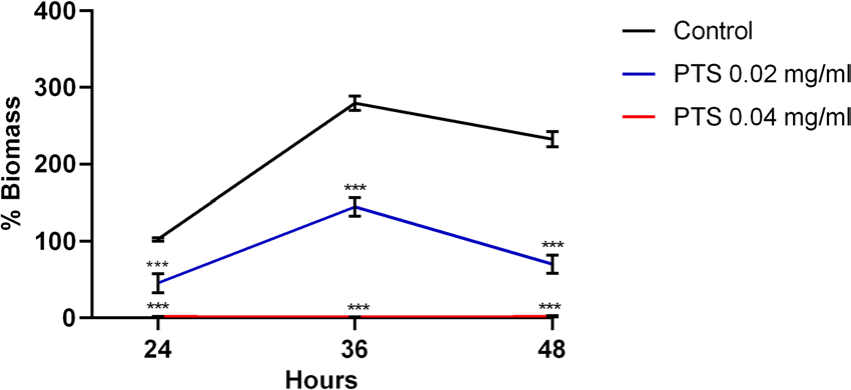
Quantification of *F. nucleatum* biofilm biomass. The amount of *F. nucleatum* biofilm formed in the presence of PTS (complexed with HPβCD) at the indicated concentrations over 48 hours was determined by crystal violet assay, and expressed as percentage biomass of control (untreated) biofilm at 24 hours. Results are expressed as the means ± SD of triplicates from three independent experiments. ***p < 0.001 compared to control group.

When dissolved in HPβCD, PTS exhibited the highest antimicrobial potency against *F. nucleatum*, with MIC and MBC values of 0.02 mg/ml and 0.04 mg/ml, respectively. This effect was attributed to PTS because HPβCD concentrations corresponding to the MIC and MBC of PTS did not affect the growth of *F. nucleatum* (Supplementary Fig. S1). Interestingly, the antimicrobial effects of PTS complexed with HPβCD against *F. nucleatum* were at least 60-fold higher compared to those of RES, OXY and PIC, which were all unable to achieve MICs even at their maximum solubility in HPβCD. In contrast, PTS dissolved in DMSO demonstrated markedly inferior antimicrobial efficacy against *F. nucleatum*, with an MIC and MBC of more than 0.15 mg/ml. Conversely, RES and OXY exhibited more potent antimicrobial effects when dissolved in DMSO, with at least 8-fold lower MICs than when complexed with HPβCD. At its MBC (0.04 mg/ml), PTS in HPβCD completely inhibited biofilm formation of *F. nucleatum* (Fig. 2). A weaker, though statistically significant, biofilm inhibition was noted at the MIC (0.02 mg/ml). As MBCs were not achievable with RES, OXY and PIC complexed with HPβCD, anti-biofilm experiments were not carried out with these compounds.

### PTS induced leakage of cellular contents

**Figure 3:**
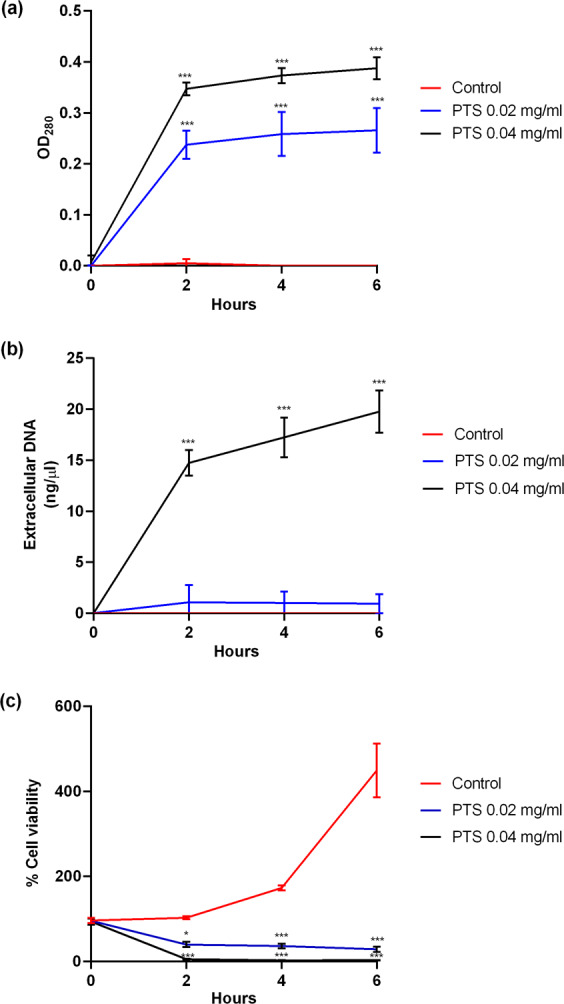
PTS triggered leakage of cellular contents. The amounts of extracellular (**a**) proteins and (**b**) nucleic acid were measured at 0, 2, 4 and 6 hours following treatment of *F. nucleatum* with PTS. (**c**) Viability of *F. nucleatum* following treatment with PTS was determined by ATP assay. Results are expressed as the means ± SD of triplicates from three independent experiments. *p < 0.05, ***p < 0.001 com*p*ared to the respective control (untreated) bacteria at the respective time points.

At the MIC of 0.02 mg/ml, PTS induced a significant leakage of bacterial proteins into the extracellular environment (Fig. 3a). At the MBC of 0.04 mg/ml, PTS induced leakage of both bacterial proteins and nucleic acids (Fig. 3a,b). This occurred most notably within the first 2 hours of *F. nucleatum* exposure to PTS. The leakage of cellular contents was noted in conjunction with a decrease in bacterial cell viability (Fig. 3c).

### Anti-inflammatory effects of PTS

**Figure 4:**
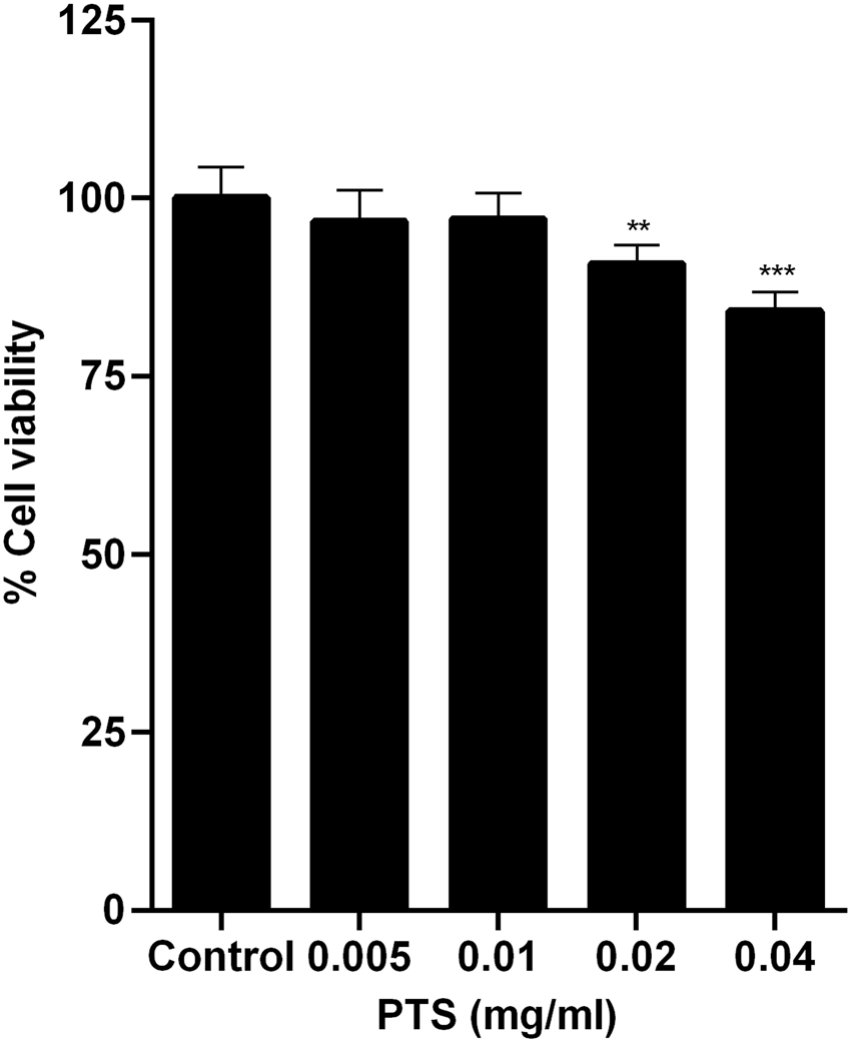
Determination of non-cytotoxic concentrations of PTS. Viability of RAW 264.7 macrophages following treatment with PTS at the indicated doses was determined by MTS assay. Cell viability was expressed as percentage of the untreated control group. Results are expressed as the means ± SD of triplicates from three independent experiments. **p < 0.01, ***p < 0.001.

To determine the anti-inflammatory efficacy of PTS in HPβCD, experiments were carried out using non-cytotoxic PTS concentrations, which were determined to be ≤0.01 mg/ml (Fig. 4).

**Figure 5:**
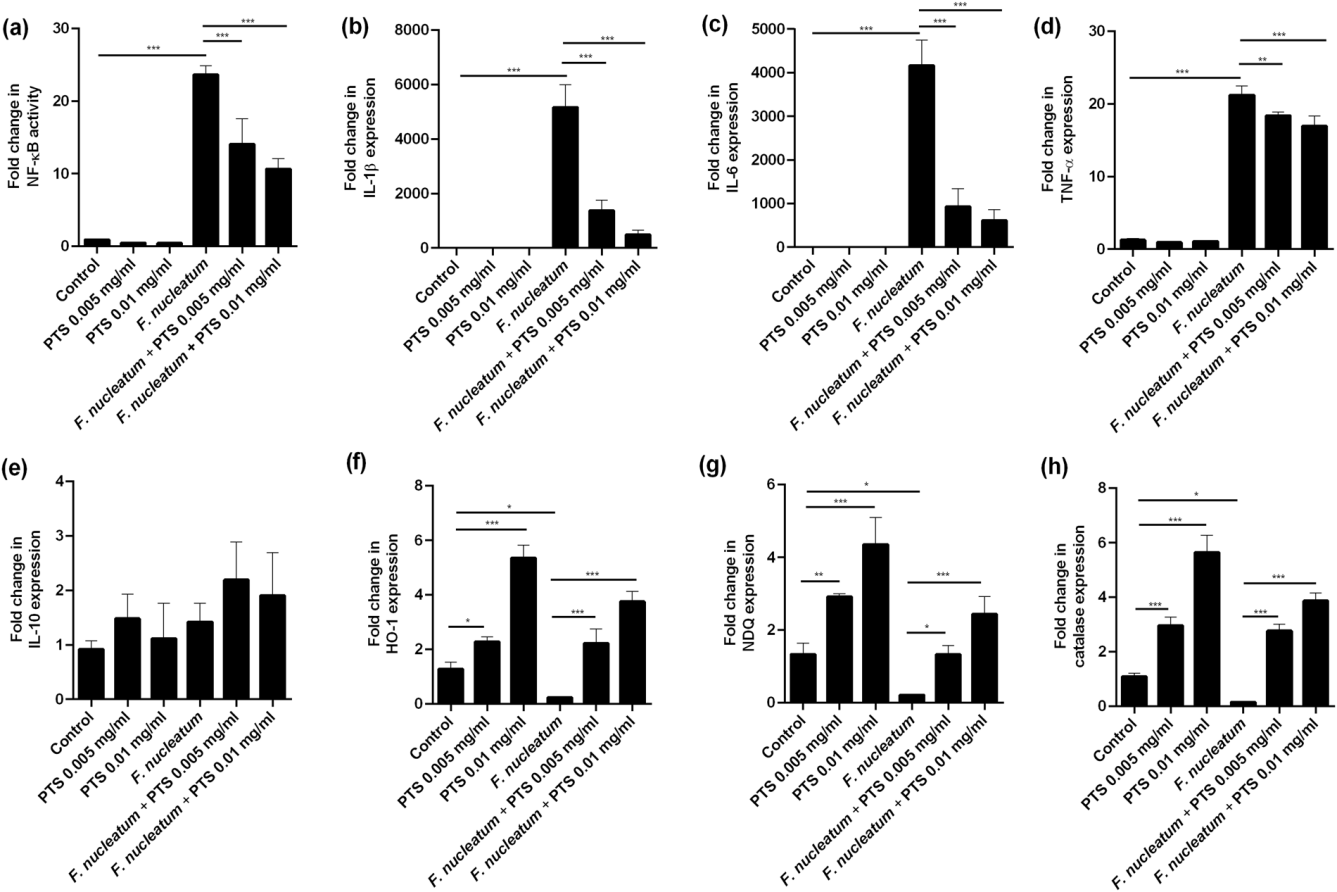
PTS elicited anti-inflammatory and antioxidant effects in macrophages. (**a**) Cellular NF-κB activity was determined by reporter assay. The expression of (**b**) IL-1β, (**c**) IL-6, (**d**) TNF-α, (**e**) IL-10, (**f**) HO-1, (**g**) NDQ, and (**h**) catalase were determined by qPCR. Results are expressed as the means ± SD of triplicates from three independent experiments. *p < 0.05, **p < 0.01, ***p < 0.001.

Stimulation of RAW 264.7 cells with *F. nucleatum* led to a pronounced increase in nuclear-factor kappa B (NF-κB) activity, which corresponded with up-regulation of interleukin (IL)-1β, IL-6 and tumour necrosis factor (TNF)-α (Fig. 5a–d). PTS treatment resulted in a dose-dependent inhibition of NF-κB activity and decreases in expression of IL-1β, IL-6 and TNF-α. However, the expression of IL-10 by challenged RAW 264.7 cells was not affected by PTS treatment (Fig. 5e). Instead, we found that PTS rescued *F. nucleatum*-induced downregulation of antioxidant gene expression, i.e. heme oxygenase 1 (HO-1), NAD(P)H dehydrogenase quinone (NDQ) and catalase gene expression in a dose-dependent manner (Fig. 5f–h).

## Discussion

The pathogenesis of periodontitis involves a complex interplay between the dysbiotic microbial community and the resultant disproportionate host immune response, resulting in a loss of homeostasis^7^. In this state of dysbiosis, an increased abundance of *F. nucleatum* further stimulates the pro-inflammatory response to its lipopolysaccharides. Virulence factors, such as adhesins, also allow *F. nucleatum* to help other bacteria colonise and persist in the biofilm^38^. Although some adjunctive host-modulation therapies are available for treatment of periodontitis, few exhibits both anti-inflammatory and antimicrobial properties, and the indiscriminate use of, for example, azithromycin and tetracyclines, is avoided due to concerns with antibiotic resistance. Thus, an alternative to these antibiotics is to seek out natural products with both antimicrobial and immune modulatory effects. In this regard, RES and its analogues have been shown to possess antimicrobial activity against various pathogens including *Vibrio cholerae, Listeria monocytogenes, Listeria innocua, Escherichia coli*, *Pseudomonas aeruginosa*^39–41^. However, these studies investigated the antimicrobial effects of these compounds when dissolved in organic solvents such as DMSO, which limits clinical translatability due to its associated cytotoxicity issues. Although HPβCD is a promising alternative solvent by virtue of its safety profile and extensive usage in pharmaceutical products, the antimicrobial properties of RES and its analogues when complexed with HPβCD remains unknown. To this end, we investigated the antibacterial and anti-inflammatory properties of these compounds against a key periopathogen (*F. nucleatum*) with HPβCD as the solubility enhancing excipient.

Interestingly, only PTS demonstrated superior antibacterial effects against *F. nucleatum* when complexed with HPβCD (as compared to when dissolved in DMSO). This is in contrast to RES, OXY and PIC, which were not able to eradicate *F. nucleatum* even at concentrations greater than 60-folds. Furthermore, PTS was found to completely inhibit *F. nucleatum* biofilm formation, which could be highly relevant in the context of periodontitis, given that biofilm reduction is a critical therapeutic aim in its management. The observation that PTS exerted a more pronounced antibacterial effect when in HPβCD (as compared to DMSO) may be explained by the difference in which the two enhance the aqueous solubility of a compound. Being a cosolvent, DMSO increases aqueous solubility in an exponential manner, whereas cyclodextrins do so in a linear fashion, especially those which form 1:1 complexes with their compounds^20^. Therefore, although organic compounds may be soluble in DMSO, they could potentially precipitate out in an aqueous environment, albeit invisible to the naked eye, resulting in reduced potency. This is especially so for PTS, which is significantly more hydrophobic than the other 3 tested compounds, owing to the presence of methoxy groups in its chemical structure (Fig. 1). In fact, because the other 3 compounds are more water soluble due to presence of hydroxy (-OH) groups, their degree of precipitation in DMSO may be negligible in the presence of solubility enhancers (such as proteins) present in the media. As a result, out of all, PTS would have the least proportion of active compound available for acting on the bacterial cells, when dissolved in DMSO.

Antimicrobials exert their effects via different mechanisms of action. For example, they may disrupt cell membranes, inhibit nucleic acid metabolism, or disrupt synthesis of important components critical for bacterial cell survival such as proteins, nucleic acids, or cell walls. Several studies in the literature^42–45^ have investigated the potential modes of action of polyphenols against various Gram positive and negative bacteria, such as *S. aureus, E. coli, P. aeruginosa* and *P. gingivalis*. These studies showed that the polyphenols act on the bacterial cell membranes, resulting in altered cell surface morphology, subsequent damage and increased cell permeability. In line with these findings, our results demonstrated that PTS likely exerted its antimicrobial properties through inducing membrane leakage, resulting in spillage of cellular contents such as DNA and proteins into the extracellular environment, which corresponded with a decrease in bacterial cell viability.

In addition to antimicrobial properties, our study also showed that PTS in HPβCD exerted immunomodulatory effects on macrophages. Macrophages have a key role in the host immune response in innate immunity and are one of the first types of immune cells to react to the bacterial challenge in the pathogenesis of periodontitis^46^. Upon stimulation, they are also capable of secreting a large range of cytokines and chemokines which interact in interconnecting networks and further drive the inflammatory response^47^.

It is interesting to note that although IL-10 is a key anti-inflammatory cytokine, PTS did not induce any changes in its expression levels. The available literature provides some evidence on the ability of RES to upregulate IL-10 expression^48,49^, thereby contributing to its anti-inflammatory properties. In our study, PTS upregulated antioxidant genes instead. The NF-κB transcription factor is redox-sensitive and is also upregulated by increased oxidative stress levels^50^. Previous studies have shown that *F. nucleatum* challenge is capable of inducing oxidative stress and production of reactive oxygen species (ROS) in macrophages^51^. The upregulation of the antioxidant pathway by PTS could reduce the amount of produced ROS and cellular oxidative stress, thus exerting indirect modulatory effects on NF-κB activation. Indeed, macrophages treated with PTS demonstrated enhanced antioxidant defence with upregulation of HO-1, NDQ and catalase, which are key molecules involved in reducing oxidative stress. NDQ and catalase are involved respectively in the scavenging of superoxides and breakdown of hydrogen peroxide, while HO-1 is a highly conserved enzyme important for heme catabolism resulting in cytoprotective effects in response to cellular oxidative stress^52,53^. This antioxidant rescue effect is in agreement with findings of recent studies investigating PTS when complexed with HPβCD, but in medical models^54,55^.

This antioxidant effect of PTS further strengthens its potential utility as an adjunctive therapy in periodontitis treatment. Oxidative stress mechanisms play a key role in periodontal tissue destruction^56^, causing ROS-mediated tissue damage directly or indirectly via redox-sensitive transcription factors such as the NF-κB signalling pathway. Clinical studies have also demonstrated associations between severe periodontitis and elevated oxidative stress^57^. Evidence from intervention studies, though still highly limited, point to the biological plausibility of improvement in periodontal outcomes resulting from nutritional interventions with a diet rich in antioxidants^58^. Furthermore, elevated oxidative stress features prominently both in the pathogenesis of type 2 diabetes and in the sequelae of AGE-RAGE (advanced glycated end-products and their receptors) interactions characteristic of the disease^59^. Thus, with results from pre-clinical studies demonstrating the anti-diabetic^60^ and anti-obesity^61^ effects of PTS treatment, PTS may also confer additional systemic benefits in periodontitis patients suffering from other co-morbidities such as diabetes and obesity. Together with promising findings on anti-oral cancer properties^62^, PTS as a functional food indeed has potential to benefit overall patient well-being. In addition, the superior pharmacokinetic profile of PTS makes it a more attractive functional food candidate than RES and its other analogues. A previous study demonstrated that PTS could reach the systemic circulation when delivered via sublingual administration, indicating its ability to efficiently penetrate the oral mucosa at very high concentrations^37^. This property further increases the potential applicability for PTS to be incorporated into chewing gums, lozenges, or some such functional food. However, pre-clinical studies in larger animal models are still needed, prior to clinical trials to confirm these potential adjunctive benefits of PTS in the clinical treatment of periodontitis. Further work is also required in terms of developing and optimising the aforementioned user-friendly dosage forms, such as chewing gums and lozenges.

In summary, the findings reported in this manuscript show that PTS, complexed with HPβCD, is capable of conferring antimicrobial, anti-inflammatory and antioxidant effects in the presence of an *F. nucleatum* bacterial challenge. Furthermore, we have also shown that complexation of PTS with HPβCD leads to increased antimicrobial potency as compared to its other analogues. Taken together, these support a focus for future research directed toward PTS, complexed with HPβCD, as a candidate nutraceutical for the adjunctive treatment of periodontitis.

## Methods

### Reagents

PTS (trans-3,5-dimethoxy-4′-hydroxystilbene, purity >99%), OXY (trans-3,5,2′,4′-tetrahydroxystilbene, purity >95%) and PIC (trans-3,5,3′,4′-tetrahydroxystilbene, purity ≥98%) were obtained from Tokyo Chemical Industry (Tokyo, Japan). RES (trans-3,5,4′-trihydroxystilbene, purity ≥99%) was purchased from Sigma-Aldrich (St. Louis, MO, USA). HPβCD (degree of substitution about 0.6) was kindly provided by Wacker (Burghausen, Germany).

### Bacterial culture

*F. nucleatum* ATCC 25586 was obtained from the American Type Culture Collection (Manassas, VA, USA). The bacterium was cultured in brain-heart infusion broth (BHI) (Acumedia, Lansing, MI, USA) supplemented with 0.5% yeast extract (Acumedia), 5 µg/ml hemin and 1 µg/ml vitamin K (Sigma-Aldrich, St. Louis, MO, USA) and incubated overnight in an anaerobic chamber (Don Whitley Scientific, Frederick, MD, USA) at 37 °C.

### Determination of minimum inhibitory concentration and minimum bactericidal concentration

The inclusion complexes were prepared by dissolving RES, OXY, PIC and PTS in 15 mM HPβCD at a concentration of 1.25 mg/ml. The same method was used for all 4 compounds. To avoid photoisomerization of the stilbenes, handling of these compounds was carried out in a dimly lit environment. MIC was determined by the broth microdilution method^63^. Briefly, a 96-well microtiter plate (Nunc, Rochester, NY) containing 100 μl of serially diluted test compound was inoculated with 100 μl of *F. nucleatum* with approximately 5 × 10^6^ colony forming units. Following 24 hours of incubation in an anaerobic chamber at 37 °C, bacterial growth was determined by measuring optical density at 600 nm using a 96-well plate reader (BioTek, Winooski, VT, USA). MBC was determined by serial dilution and plating of *F. nucleatum* on tryptic soy agar supplemented with 5% sheep blood (Oxoid, Hampshire, UK). A parallel experiment with the test compounds dissolved in DMSO was also performed, where RES, OXY, PIC and PTS were dissolved in 1% DMSO at a concentration of 0.15 mg/ml.

### Effect of PTS on biofilm formation

*F. nucleatum* biofilm was grown in the presence or absence of PTS in 24-well plates (Nunc). The plates were incubated anaerobically at 37 °C for 24, 36 or 48 h. Culture media was refreshed every 24 h. The amount of *F. nucleatum* biomass was quantified by crystal violet assay. Briefly, culture supernatant was removed, and biofilm was washed twice with phosphate-buffered saline (PBS) to remove planktonic bacteria. The biofilm was fixed with 100% methanol, before removal and drying. Biofilm was stained with 0.1% crystal violet. Excess crystal violet was removed with copious washing with distilled water before dissolving the crystal violet stains in 33% acetic acid. Optical density at 580 nm was read using a 96-well plate reader (BioTek).

### Determination of cellular content leakage

*F. nucleatum* was cultured anaerobically at 37 °C in the presence or absence of PTS. Bacterial culture supernatant was removed at the required time-point. The quantities of nucleic acids and proteins in the culture supernatant were determined by measuring optical density at 260 nm and 280 nm, respectively. In parallel, bacterial cell viability was determined by the ATP assay using the BacTiter-Glo reagent (Promega, Madison, WI, USA).

### Cell culture

RAW 264.7 macrophages were obtained from the ATCC. The cells were cultured in DMEM supplemented with 10% heat-inactivated fetal bovine serum (FBS) (Life Technologies, Rockville, MD, USA) and incubated at 37 °C with 5% CO_2_. For analysis of cellular NF-κB activity, RAW 264.7 cells stably expressing the NF-κB-secreted alkaline phosphatase (SEAP) reporter was used^64^. The SEAP activity in the culture medium was measured with a Phospha-Light assay kit (Life Technologies), according to manufacturer’s instructions.

### Cell viability assay

Viability of macrophages following treatment with PTS was determined by the MTS assay using the CellTiter 96 Aqueous One Solution Cell Proliferation Assay kit (Promega) following the manufacturer’s protocol.

### RNA extraction and qPCR

Total RNA was extracted using the RNeasy Mini Kit (Qiagen, Valencia, CA, USA) with on-column DNaseI treatment to remove any contaminating DNA present in the RNA samples. Reverse transcription (RT) was performed using iScript Reverse Transcription SuperMix (BioRad, Hercules, CA, USA). The expressions of IL-1β, IL-6, TNF-α, HO-1, NDQ and catalase were determined by quantitative PCR (qPCR). The final volume of reaction mixture comprised 10 µl of iTaq Universal SYBR Green SuperMix (BioRad), and 10 pmol each of the respective forward and reverse primers. Thermocycling and signal detection were carried out using a CFX Connect Real-Time Detection System (BioRad). The expression of the respective target genes was normalised to the relative abundance of the housekeeping gene β-actin and expressed as fold change using the ΔΔC_T_ method. The sequences of the primers used are listed in Table 2.

**Table 2:** Sequences of primers used.

Gene	Forward primer sequence	Reverse primer sequence
β-actin	5′- GGCTGTATTCCCCTCCATCG	5′- CCAGTTGGTAACAATGCCATGT
IL-1β	5′- CAACCAACAAGTGATATTCTCCATG	5′- GATCCACACTCTCCAGCTGCA
IL-6	5′- CTGCAAGAGACTTCCATCCAGTT	5′- GAAGTAGGGAAGGCCGTGG
TNF-α	5′- CATCTTCTCAAAATTCGAGTGACAA	5′- TGGGAGTAGACAAGGTACAACCC
HO-1	5′- AAGCCGAGAATGCTGAGTTCA	5′- GCCGTGTAGATATGGTACAAGGA
NDQ	5′- TCTGCAGCTTCCAGCTTCTTG	5′- TATCCTTCCGAGTCATCTCTAGCA
Catalase	5′- AGCGACCAGATGAAGCAGTG	5′- TCCGCTCTCTGTCAAAGTGTG

### Statistical analysis

All experiments were carried out in triplicate and repeated 3 independent times. Statistical analyses were performed using a one-way analysis of variance with a post hoc Tukey multiple comparison test (GraphPad Software Inc., USA). All results were considered statistically significant at *p* < 0.05.

## Supplementary information

Supplementary Information.
