# Supplementary material for: Pterostilbene complexed with cyclodextrin exerts antimicrobial and anti-inflammatory effects
Source: Sci Rep. 2020 Jun 3;10:9072. doi: 10.1038/s41598-020-66031-8 (PMC7271226; doi:10.1038/s41598-020-66031-8)

**Pterostilbene complexed with cyclodextrin exerts**

**antimicrobial and anti-inflammatory effects**

Yi Rong Ivan Lim^1^, Philip M. Preshaw^1^, Lum Peng Lim^1^, Marianne Meng Ann Ong^2^,

Hai-Shu Lin^3^ and Kai Soo Tan^1*^

^1^Faculty of Dentistry, National University of Singapore, Singapore

^2^National Dental Centre, Singapore

^3^Department of Pharmacy, Faculty of Science, National University of Singapore, Singapore

^*^Corresponding author:

Kai Soo Tan, PhD

Faculty of Dentistry

National University of Singapore

9 Lower Kent Ridge Road,

National University Centre for Oral Health

Singapore 119085

Email: denkst@nus.edu.sg

Tel: +65-6772-8842

Fax: +65-6774-5701

**Supplementary Information**

Supplementary Figure S1. Effect of HPβCD alone on planktonic *F. nucleatum.* Growth of planktonic *F. nucleatum* was determined by measuring optical density at 600 nm. At the indicated HPβCD concentrations (0.23 and 0.47 mM) corresponding to MIC and MBC of PTS respectively, HPβCD did not exert any statistically significant inhibitory effect on planktonic *F. nucleatum*. Results are expressed as the means ± SD of triplicates from three independent experiments.


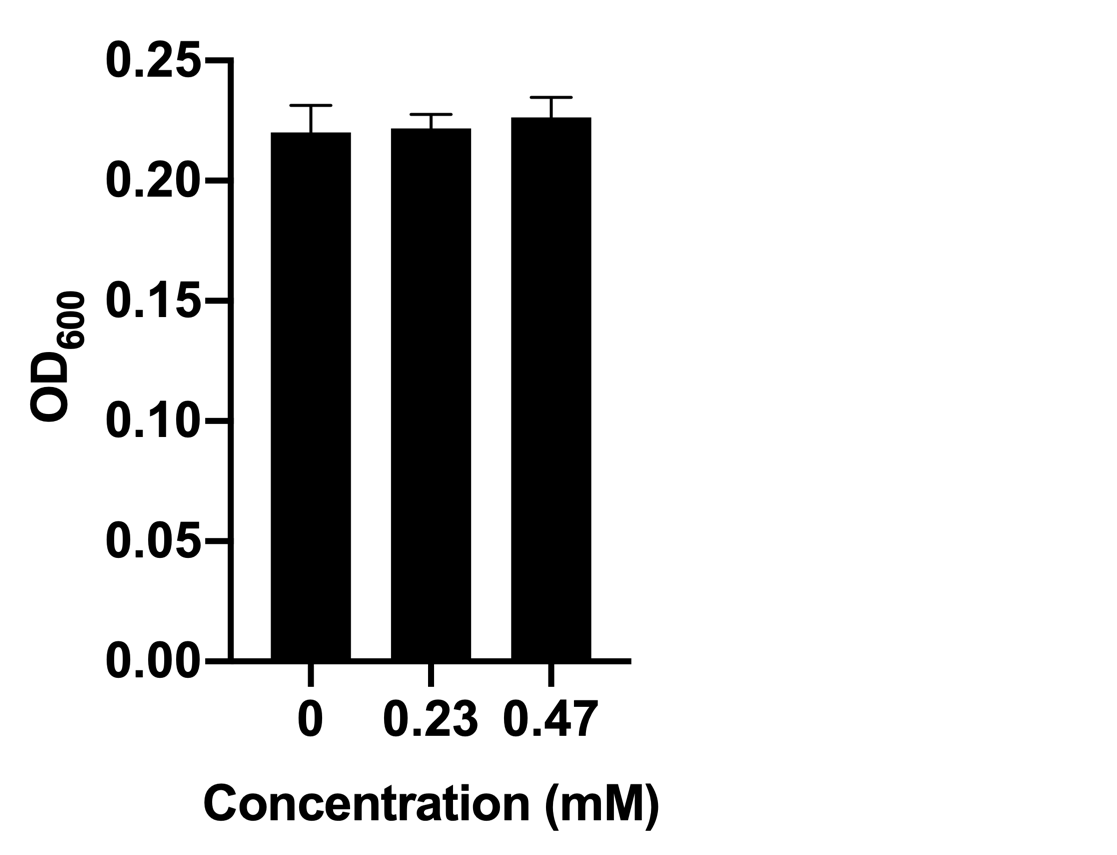

Supplement: Supplementary file 1 — Supplementary Information. [file 41598_2020_66031_MOESM1_ESM.docx]
